# Dasatinib Reduces Lung Inflammation and Fibrosis in Acute Experimental Silicosis

**DOI:** 10.1371/journal.pone.0147005

**Published:** 2016-01-20

**Authors:** Fernanda Ferreira Cruz, Lucas Felipe Bastos Horta, Lígia de Albuquerque Maia, Miquéias Lopes-Pacheco, André Benedito da Silva, Marcelo Marco Morales, Cassiano Felippe Gonçalves-de-Albuquerque, Christina Maeda Takiya, Hugo Caire de Castro-Faria-Neto, Patricia Rieken Macedo Rocco

**Affiliations:** 1 Laboratory of Pulmonary Investigation, Carlos Chagas Filho Institute of Biophysics, Federal University of Rio de Janeiro, Rio de Janeiro, Brazil; 2 Laboratory of Cellular and Molecular Physiology, Carlos Chagas Filho Institute of Biophysics, Federal University of Rio de Janeiro, Rio de Janeiro, Brazil; 3 Laboratory of Immunopharmacology, Oswaldo Cruz Institute, Fiocruz, Rio de Janeiro, Brazil; 4 Laboratory of Cellular Pathology, Carlos Chagas Filho Institute of Biophysics, Federal University of Rio de Janeiro, Rio de Janeiro, Brazil; University of Rochester Medical Center, UNITED STATES

## Abstract

Silicosis is an occupational lung disease with no effective treatment. We hypothesized that dasatinib, a tyrosine kinase inhibitor, might exhibit therapeutic efficacy in silica-induced pulmonary fibrosis. Silicosis was induced in C57BL/6 mice by a single intratracheal administration of silica particles, whereas the control group received saline. After 14 days, when the disease was already established, animals were randomly assigned to receive DMSO or dasatinib (1 mg/kg) by oral gavage, twice daily, for 14 days. On day 28, lung morphofunction, inflammation, and remodeling were investigated. RAW 264.7 cells (a macrophage cell line) were incubated with silica particles, followed by treatment or not with dasatinib, and evaluated for macrophage polarization. On day 28, dasatinib improved lung mechanics, increased M2 macrophage counts in lung parenchyma and granuloma, and was associated with reduction of fraction area of granuloma, fraction area of collapsed alveoli, protein levels of tumor necrosis factor-α, interleukin-1β, transforming growth factor-β, and reduced neutrophils, M1 macrophages, and collagen fiber content in lung tissue and granuloma in silicotic animals. Additionally, dasatinib reduced expression of iNOS and increased expression of arginase and metalloproteinase-9 in silicotic macrophages. Dasatinib was effective at inducing macrophage polarization toward the M2 phenotype and reducing lung inflammation and fibrosis, thus improving lung mechanics in a murine model of acute silicosis.

## Introduction

Silicosis is an occupational disease caused by inhalation of crystalline silica particles, which triggers a persistent inflammatory cascade that leads to progressive lung fibrosis and subsequent respiratory failure due to deterioration of lung function and reduction in gas exchange area [1]. Although therapy for silicosis includes a variety of drugs and non-pharmacological interventions, there is still a pressing need for new therapeutic approaches as no current therapy is able to effectively reduce disease progression or reverse lung fibrosis [2,3].

Dasatinib (DAS; Bristol-Myers Squibb, New York, NY, USA), which has been widely studied for the treatment of cancer [4,5], is a second-generation oral multitarget inhibitor of several tyrosine kinases, including Abl and Bcr-Abl family members, Src and Btk family members, c-Kit, PDGFR, and Eph receptors [6,7]. Its targets include several receptors associated with the regulation of a wide variety of pathways involved in physiological cell functions and pathophysiological processes such as the recruitment and activation of inflammatory cells [8–14] and fibrosis [15,16]. Thus, inhibition of tyrosine kinases receptors may reduce inflammation and slow the progression of pulmonary fibrosis.

We hypothesized that dasatinib would attenuate pulmonary fibrosis, ameliorate inflammatory responses, and improve lung function in experimental acute silicosis. For this purpose, we investigated the potential efficacy and mechanisms of dasatinib in the treatment of silica-induced lung fibrosis.

## Material and Methods

This study was approved by the Ethics Committee of the Health Sciences Centre, Federal University of Rio de Janeiro (CEUA-CCS-019). All animals received humane care in compliance with the “Principles of Laboratory Animal Care” formulated by the National Society for Medical Research and the U.S. National Research Council “Guide for the Care and Use of Laboratory Animals”, and all efforts were made to minimize suffering.

### Animal Preparation and Experimental Protocol

**Fig 1:**
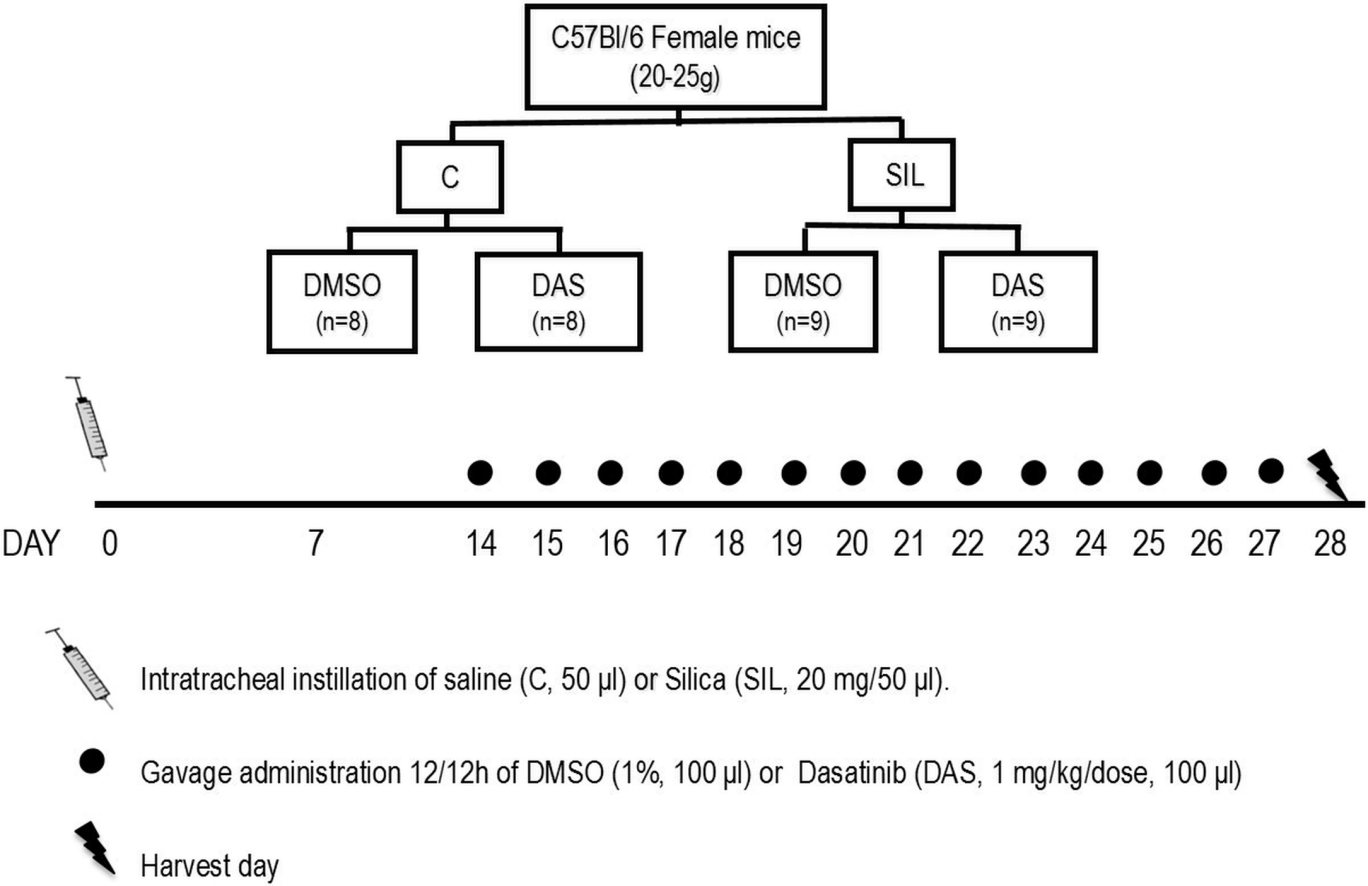
Study design. Thirty-four C57BL6 female mice (8–12 weeks, 20–25 g) were divided into two groups: control group (C, n = 16) instilled with sterile saline (50 μL, intratracheally [i.t.]) and silicosis group (SIL, n = 18), instilled with silica particle (20mg in 50 μL saline, i.t.). Fourteen days after disease induction, the animals were randomized to receive a solution of dimethyl sulfoxide (DMSO 1% in saline solution, 100 μL, oral gavage, n = 8/9) or dasatinib (DAS 1 mg/kg body weight in DMSO 1%, 100 μL, oral gavage, n = 8/9).

Fifty-two C57BL/6 female mice (weight: 20–25 g, age 8–12 weeks) were assigned to two main groups: control (C) and silicosis (SIL). In group SIL, mice received silica particle suspension (20 mg in 50μL saline, intratracheally [i.t.]), while group C received saline (50 μL, i.t.) using the same protocol. Fourteen days after administration of silica or saline, animals were further randomized to receive dimethyl sulfoxide (DMSO 1% in saline solution, 100 μL, oral gavage) or dasatinib (DAS 1 mg/kg body weight in DMSO 1%, 100 μL, oral gavage) during 14 days (Fig 1). Additionally, another group of animals was treated with saline (SAL, 100 μL) by oral gavage for 14 days. These animals were subsequently compared to those that received DMSO, aiming to evaluate whether DMSO *per se* might have any effects on lung morphofunction (Tables A-C in S1 File).

### Lung Mechanics

Twenty-four hours after the last dose, the animals were sedated (diazepam 1 mg i.p.), anesthetized (thiopental sodium 20 mg/kg i.p.), tracheotomized, paralyzed (vecuronium bromide, 0.005 mg/kg i.v.), and ventilated with a constant flow ventilator (Samay VR15; Universidad de la Republica, Montevideo, Uruguay) set to the following parameters: respiratory rate 100 breaths/min, tidal volume (V_T_) 0.2 mL, and fraction of inspired oxygen (FiO_2_) 0.21. The anterior chest wall was surgically removed and a positive end-expiratory pressure of 2 cm H_2_O applied. Airflow and tracheal pressure (Ptr) were measured. In an open chest preparation, Ptr reflects transpulmonary pressure (P_L_). After a 10-min ventilation period, static lung elastance (*E*st,L), and lung resistive (Δ*P*1,L) and viscoelastic/inhomogeneous pressures (Δ*P*2,L) were measured using the end-inflation occlusion method [17]. All data were analyzed using ANADAT software (RHT-InfoData, Inc., Montreal, Quebec, Canada). All experiments lasted less than 15 min.

### Histology

Immediately after determination of lung mechanics, a laparotomy was performed and heparin (1000 IU) was injected in the vena cava. The trachea was clamped at end-expiration (PEEP = 2 cmH_2_O), and the vena cava and abdominal aorta were sectioned, leading to a massive hemorrhage and euthanasia by terminal bleeding. The left lung was then removed, quickly frozen by immersion in liquid nitrogen, fixed with Carnoy’s solution and paraffin-embedded. Four 4-μm-thick slices per lung were cut and stained with hematoxylin-eosin. Lung morphometry analysis was performed with an integrating eyepiece with a coherent system consisting of a grid with 100 points and 50 lines of known length coupled to a conventional light microscope (Olympus BX51, Olympus Latin America-Inc., Sao Paulo, Brazil). The fractional area of lung occupied by collapsed or normal alveoli, or hyperinflated structures (> 120 μm) were determined by the point-counting technique [18] across 10 random, non-coincident microscopic fields at 200× magnification. Briefly, points falling on collapsed, normal pulmonary areas or hyperinflated structures were counted and divided by the total number of points in each microscopic field [18]. Additionally, the fractional area of granuloma was determined in 20 random non-coincident microscopic fields, at 400× magnification [2,19]. Neutrophils, mononuclear cells, and total cells in the alveolar septa and granuloma were evaluated at 1,000× magnification and determined by the point-counting technique. Collagen fibers (Picrosirius polarization method) were computed in the alveolar septa and granuloma at 400× magnification using Image-Pro Plus 6.3 software (Media Cybernetics, Silver Spring, MD, USA) [2,20]. Bronchi and blood vessels were carefully avoided during the measurements. The area occupied by fibers was determined by digital densitometric recognition. The results were expressed as the fractional area occupied by collagen fibers in the alveolar septa or in granuloma.

### Immunohistochemistry for Macrophages

Immunohistochemistry for total macrophages and macrophage subpopulations (M1 and M2 phenotypes) in lung tissue was performed using rat anti–mouse F4/80 monoclonal antibody (catalog MCA497G, 1:50 dilution; AbD Serotec, Bio-Rad Laboratories), inducible nitric oxide synthase (iNOS) rabbit anti-mouse polyclonal antibody (M1, catalogue no. Rb-1711, 1:100 dilution, Thermo Scientific), and arginase-1 rabbit anti-mouse polyclonal antibody (M2, catalog no. sc-20150, 1:10 dilution, Santa Cruz Biotechnology) [19,21].

Paraffin-embedded tissue sections (4 μm) were dewaxed and, after rehydration, subjected to heat-mediated antigen retrieval with citrate buffer 10 mM (pH = 6.0). Endogenous peroxidase activity was inhibited in 70% hydrogen peroxide solution in methanol. Non-specific immunoglobulin binding was blocked by 10% bovine serum albumin in phosphate saline buffer (pH = 7.4), before primary antibody incubation. Antibodies were revealed with biotinylated secondary antibody (Histofine mouse Max PO anti-rat and anti-rabbit, Nichirei Biosciences, Tokyo, Japan). Detection was done with peroxide and the chromogen substrate diaminobenzidine (catalog no. K3468, Dakocytomation). Slides were counterstained with Giemsa.

Analysis was performed in 30 images of high-power fields (400× magnification) per slide, manually selected using a light microscope (Nikon Eclipse 400; Nikon Instruments, Tokyo, Japan), and captured with an Evolution VF Color Cooled 12-bit digital camera (Media Cybernetics, Silver Spring, MD, USA). The areas occupied by nucleated macrophages and cells with positive staining for the phenotype marker in each tissue area were then measured and divided by tissue area using Image-Pro Plus 6.3 software and expressed as fractional area occupied by positive cells.

### Enzyme-Linked Immunosorbent Assay (ELISA)

Levels of interleukin (IL)-1β, tumor necrosis factor (TNF)-α and transforming growth factor (TGF)-β were quantified by ELISA in the lung homogenate. Lung tissue was homogenized in lysis buffer (PBS 1×, triton X 0.01%, 1× Roche protease inhibitor cocktail (Roche Diagnostic, Mannheim, Germany]) using a glass Potter homogenizer with Teflon piston. The total amount of cytokines was quantified according to the manufacturer’s protocol (Duo Set, R&D Systems, Minneapolis, MN, USA) and normalized to the total protein content quantified by Bradford’s reagent (Sigma-Aldrich, St Louis, MO, USA).

### *In vitro* Analysis of Macrophages

RAW 264.7 cells, a mouse peritoneal macrophage cell line, obtained from American Type Culture Collection (Rockville, MD) were maintained in culture, using Dulbecco’s Modified Eagle Medium (DMEM)–High Glucose, supplemented with 10% fetal bovine serum, 1,000 U/mL penicillin/streptomycin, 2mM L-glutamine (Invitrogen, Life Technologies Grand Isle, NY). Cells were plated in six-well plates (10^6^ cells/well) for 48 hours. The medium was then replaced with fresh medium, and cells were exposed to silica particles (100 μg per mL of medium) for 24 hours [22] or left incubated with regular medium. Supernatant was then removed; cells were washed with 1× PBS, and then incubated with dasatinib (100 ng/mL medium) or regular medium for 24 hours. Once again, supernatant was removed, cells washed with PBS, lifted using 2.5% Trypsin/EDTA (Invitrogen Life Technologies Grand Isle, NY) and pelleted by centrifugation (600 × *g* for 5 min).

A quantitative real-time reverse transcription (RT) polymerase chain reaction (PCR) was performed to measure mRNA expression of iNOS, arginase, metalloproteinase (MMP)-9, and caspase 3. Cells were lysed for RNA extraction through the RNeasy Plus Mini Kit (Qiagen, Valencia, CA, USA) according to the manufacturer’s recommendations. The total RNA concentration was measured by spectrophotometry in Nanodrop ND-1000. First-strand cDNA was synthesized from total RNA using an M-MLV Reverse Transcriptase Kit (Invitrogen). Relative mRNA levels were measured with a SYBR Green detection system using ABI 7500 real-time PCR (Applied Biosystems, Foster City, CA). All samples were measured in triplicate. The relative level of each gene was calculated as the ratio of the study gene to the control gene (acidic ribosomal phosphoprotein P0 [36β4]) and given as the fold change relative to RAW cells incubated with regular medium. The following PCR primers were used: iNOS forward CTTCAGGTATGCGGTATTGG and reverse 5´ CAT GGT GAA CAC GTT CTT GG; Arginase-1: forward 5' GCT CAG GTG AAT CGG CCT TTT-3' and reverse 5' TGG CTT GCG AGA CGT AGA C-3'; MMP-9 forward 5'-AGT CCG GCA GAC AAT CCT T-3' and reverse 5'-CCC TGT AAT GGG CTT CCT C-3'; caspase-3 forward 5'-TAC CGG TGG AGG CTG ACT-3' and reverse 5'- GCT GCA AAG GGA CTG GAT-3'; 36β4 forward 5'-CAA CCC AGT TCT GGA GAA AC-3' and reverse 5'-GTT CTG AGC TCC CAC AGTGA-3'.

### Statistical Analysis

The normality of data (Kolmogorov-Smirnov test with Lilliefors’ correction) and the homogeneity of variances (Levene median test) were tested. Parametric data are expressed as mean ± SD. Differences between groups were evaluated by one-way ANOVA followed by Bonferroni’s test. Nonparametric data were analyzed using ANOVA on ranks followed by Dunn’s post hoc test. All tests were performed using GraphPad Prism v6.0 statistical software package (GraphPad Software, La Jolla, California, USA). Significance was established at p < 0.05.

## Results

The mortality rate of animals with acute silicosis was 50% before treatment was initiated. The remaining 18 mice were allocated, with 9 receiving dasatinib and 9 receiving DMSO. No animal died after therapy with DMSO or dasatinib. No significant differences in lung mechanics and inflammation, fraction area of granuloma, and collagen fiber content were observed between the saline and DMSO groups (Tables A-C in S1 File).

### Lung Mechanics

**Fig 2:**
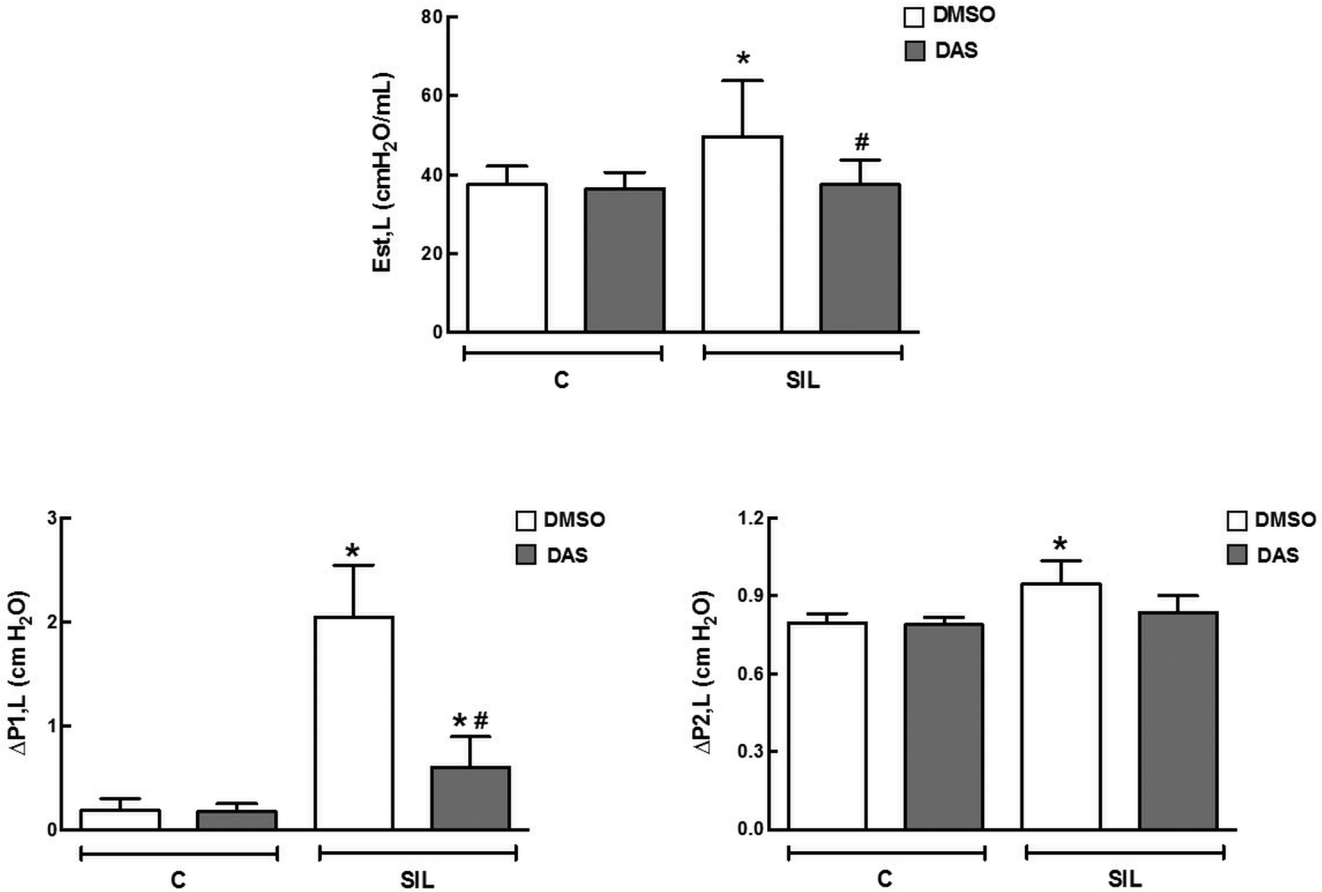
Lung mechanics. Lung static elastance (*Est*,*L*). Resistive pressure (ΔP1) and viscoelastic pressure (ΔP2). White bars: DMSO; gray bars: DAS. Values are means (± SD) of 8–9 animals per group. *Significantly different from C-DMSO group (*p <* 0.05). #DMSO *vs*. DAS (*p <* 0.05).

Tidal volume and airflow did not differ among groups. Est,L, ΔP1,L, and ΔP2,L were similar in C-DMSO and C-DAS groups. Est,L, ΔP1,L, and ΔP2,L were higher in SIL-DMSO animals than in the C-DMSO group (32%, 995%, and 19%, respectively). In the SIL-DAS group, all mechanical parameters were reduced compared to SIL-DMSO; however, ΔP1,L remained higher than C-DAS group (Fig 2).

### Lung Morphometry and Inflammation

**Fig 3:**
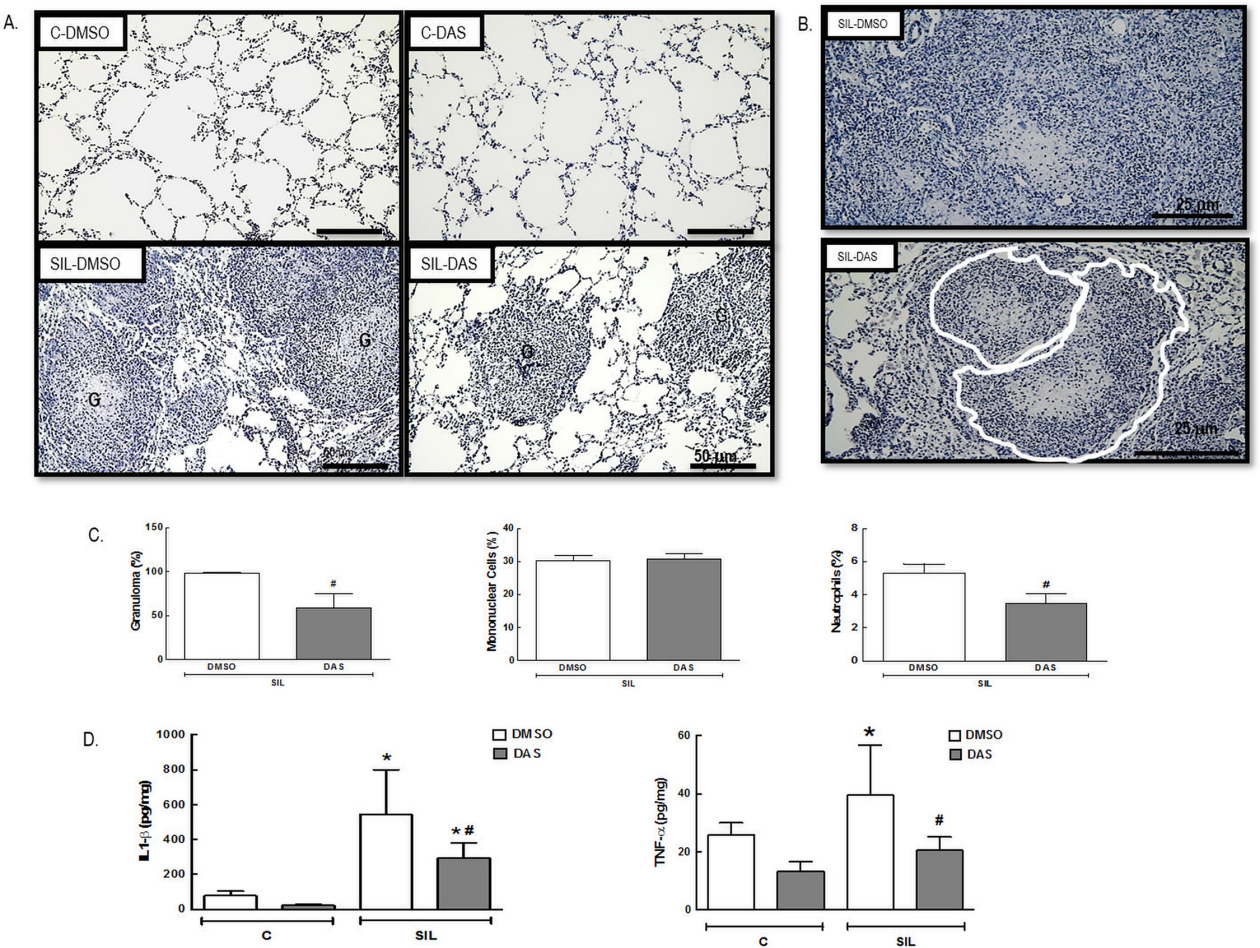
Lung and granuloma morphometry and inflammation. (A) Representative photomicrographs (light microscopy) of lung parenchyma, stained by hematoxylin and eosin, of C-DMSO, C-DAS, SIL-DMSO, SIL-DAS. G: granuloma. 100× magnification. Scale bars = 50μm. (B) Representative photomicrographs (light microscopy) of granuloma of SIL-DMSO and SIL-DAS. Red lines show that granuloma appears to disintegrate. 200× magnification. Scale bars = 25μm. (C) Granuloma fraction (%). Granuloma cellularity—Mononuclear cells and neutrophils. All values were computed in 10 random, non-coincident fields per mice. Values are means (± SD) of 8–9 animals/group. *Significantly different from C-DMSO (*p <* 0.05) ^#^Significantly different from SIL-DMSO (*p <* 0.05). (D) Lung tissue protein levels of IL-1β and TNF-α. White bars: DMSO; gray bars: DAS. Values are means (± SD) of 8–9 animals per group. *Significantly different from C-DMSO group (*p* < 0.05). ^#^DMSO *vs*. DAS (*p <* 0.05).

In the SIL-DMSO group, we observed granulomatous nodules with infiltration of neutrophils and mononuclear cells, mainly macrophages (Fig 3). Moreover, there were increased areas of alveolar collapse (214%) and cell infiltration (86% neutrophils, 66% mononuclear) in lung parenchyma (Table 1).

**Table 1:** Lung Morphometry and Differential Cell Counts.

GROUPS	C		SIL	
	DMSO	DAS	DMSO	DAS
**Normal (%)**	91.7 ± 4.8	93.1 ± 5.5	72.8 ± 4.4*	88.3 ± 3.7^#^
**Collapse (%)**	8.3 ± 4.8	6.9 ± 5.5	26.1 ± 5.6*	11.0 ± 3.9^#^
**Hyperinflation (%)**	0.0 ±0.0	0.0 ± 0.0	1.2± 1.9	0.7±1.6
**Neutrophils (%)**	2.9 ± 0.6	4.4 ± 1.1	5.4 ± 3.2*	3.9 ± 1.1^#^
**Mononuclear Cells (%)**	19.1 ± 4.4	21.0 ± 4.0	31.7 ± 7.0*	27.0 ± 3.9*
**Total Cells (%)**	22.0 ± 4.7	25.4 ± 3.8	37.1 ± 6.8*	30.9 ± 3.7*

Fraction area of normal alveoli, collapsed alveoli, lung hyperinflation, neutrophils, mononuclear and total cells. Values are means ± SD of 8–9 animals/group.

*Significantly different from C-DMSO (*p <* 0.05).

#Significantly different from SIL-DMSO (*p <* 0.05).

Photomicrographs show that granuloma appeared to be disintegrating over time after treatment with dasatinib (Fig 3). The area of collapsed alveoli, the fraction area of granuloma and neutrophils in lung parenchyma and granuloma were reduced in SIL-DAS compared to SIL-DMSO (Table 1; Fig 3).

Protein levels of IL-1βand TNF-α increased (596% and 53%, respectively) in lung tissue in SIL-DMSO compared to C-DMSO animals. Dasatinib minimized the expression of both cytokines (45.7% and 47.9%, respectively) (Fig 3).

**Fig 4:**
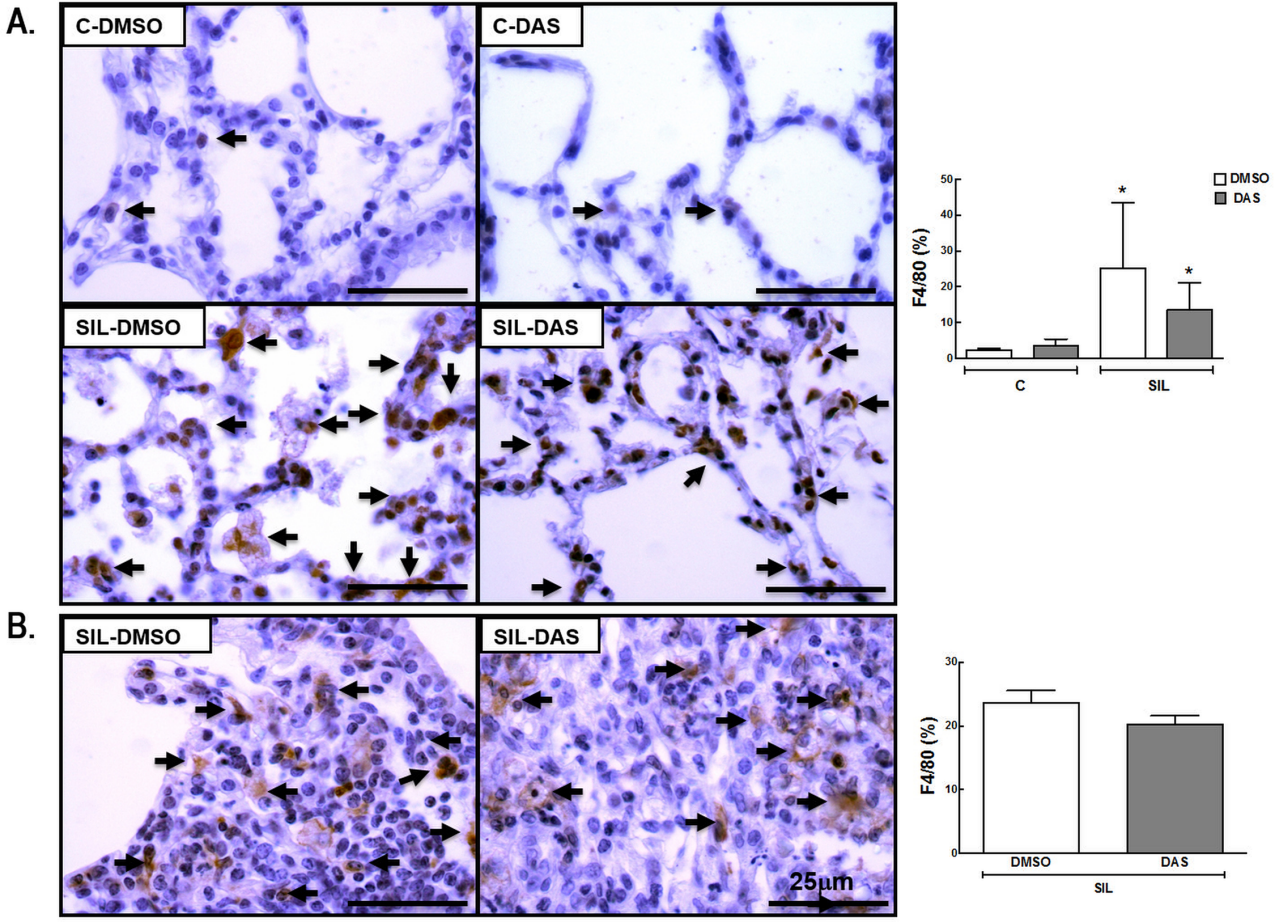
Representative immunohistochemistry photomicrographs and quantification of total macrophages (F4/80 positive cells). (A) Lung parenchyma, C-DMSO, C-DAS, SIL-DMSO, SIL-DAS. (B) Silicotic granuloma, SIL-DMSO, SIL-DAS. Arrows: F4/80 positive cells. White bars: DMSO; gray bars: DAS. Values are means (± SD) of 6 animals per group. *Significantly different from C-DMSO group (*p* < 0.05). ^#^DMSO *vs*. DAS (*p <* 0.05).

**Fig 5:**
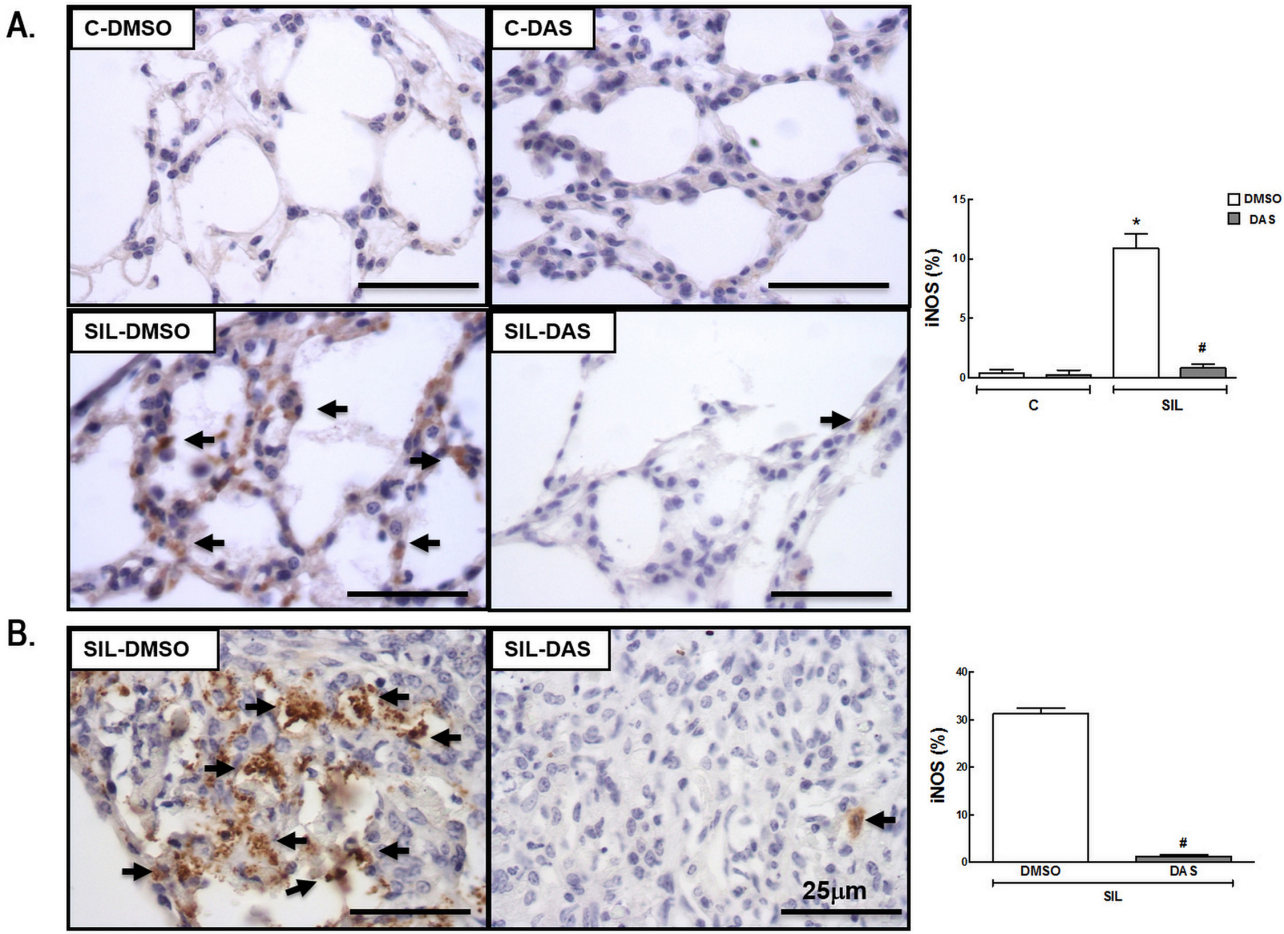
Representative immunohistochemistry photomicrographs and quantification of subtype M1 macrophages (iNOS-positive cells). (A) Lung parenchyma, C-DMSO, C-DAS, SIL-DMSO, SIL-DAS. (B) Silicotic granuloma, SIL-DMSO, SIL-DAS. Arrows: iNOS-positive cells; white bars: DMSO; gray bars: DAS. Values are means (± SD) of 6 animals per group. *Significantly different from C-DMSO group (*p* < 0.05). ^#^DMSO *vs*. DAS (*p <* 0.05).

**Fig 6:**
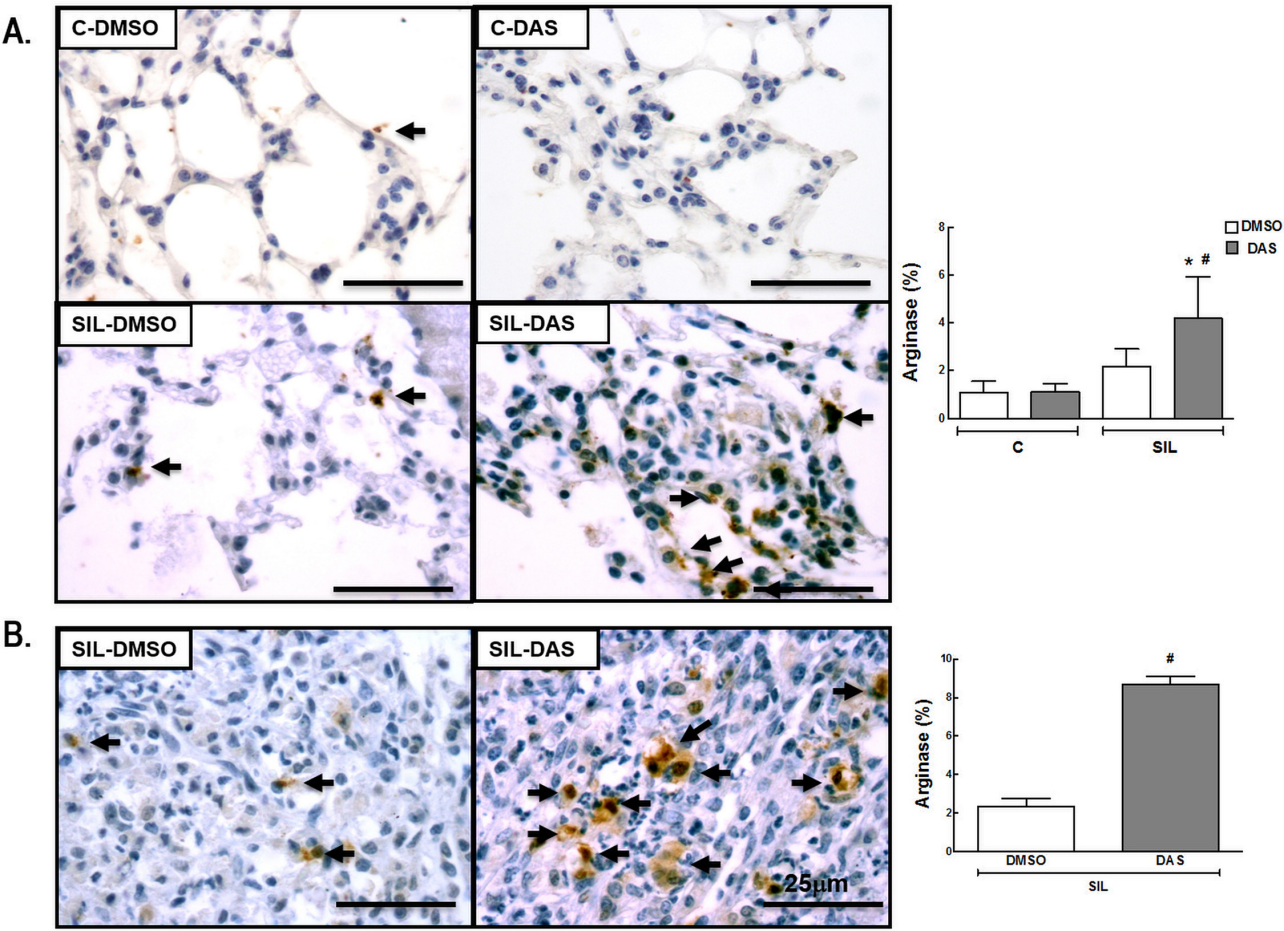
Representative Immunohistochemistry photomicrographs and quantification of subtype M2 macrophages (arginase-positive cells). (A) Lung parenchyma, C-DMSO, C-DAS, SIL-DMSO, SIL-DAS. (B) Silicotic granuloma, SIL-DMSO, SIL-DAS. Arrows: arginase-positive cells; white bars: DMSO; gray bars: DAS. Values are means (± SD) of 6 animals per group. *Significantly different from C-DMSO group (*p* < 0.05). ^#^DMSO *vs*. DAS (*p <* 0.05).

Macrophages are considered the main cells in the pathophysiology of silicosis. They can be activated by a variety of extracellular signals to polarize into M1 macrophages (associated with antimicrobial response and inflammation) or M2 macrophages (associated with wound healing and resolution of inflammation). The total number of macrophages (F4/80 positive cells) and the M1 (iNOS positive cells) and M2 (arginase-1 positive cells) subpopulations were quantified. The number of macrophages in alveolar septa was higher (1,036%) in the SIL-DMSO group than in the C-DMSO group (Fig 4). Dasatinib did not reduce the number of macrophages either in lung parenchyma or in granuloma (Fig 4, Table D in S1 File). The number of M1 macrophages was increased in lung parenchyma (1,160%) in SIL-DMSO compared to C-DMSO animals, and reduced in SIL-DAS in lung parenchyma (92.3%) and granuloma (96.8%) (Fig 5, Table E in S1 File). No significant difference was observed in the number of M2 macrophages between the SIL-DMSO and C-DMSO groups. However, dasatinib increased the number of M2 macrophages in lung parenchyma (712.3%) and granuloma (336.5%) in animals with acute silicosis (Fig 6, Table F in S1 File).

### Lung Fibrosis (Collagen Fibers and TGF-β)

**Fig 7:**
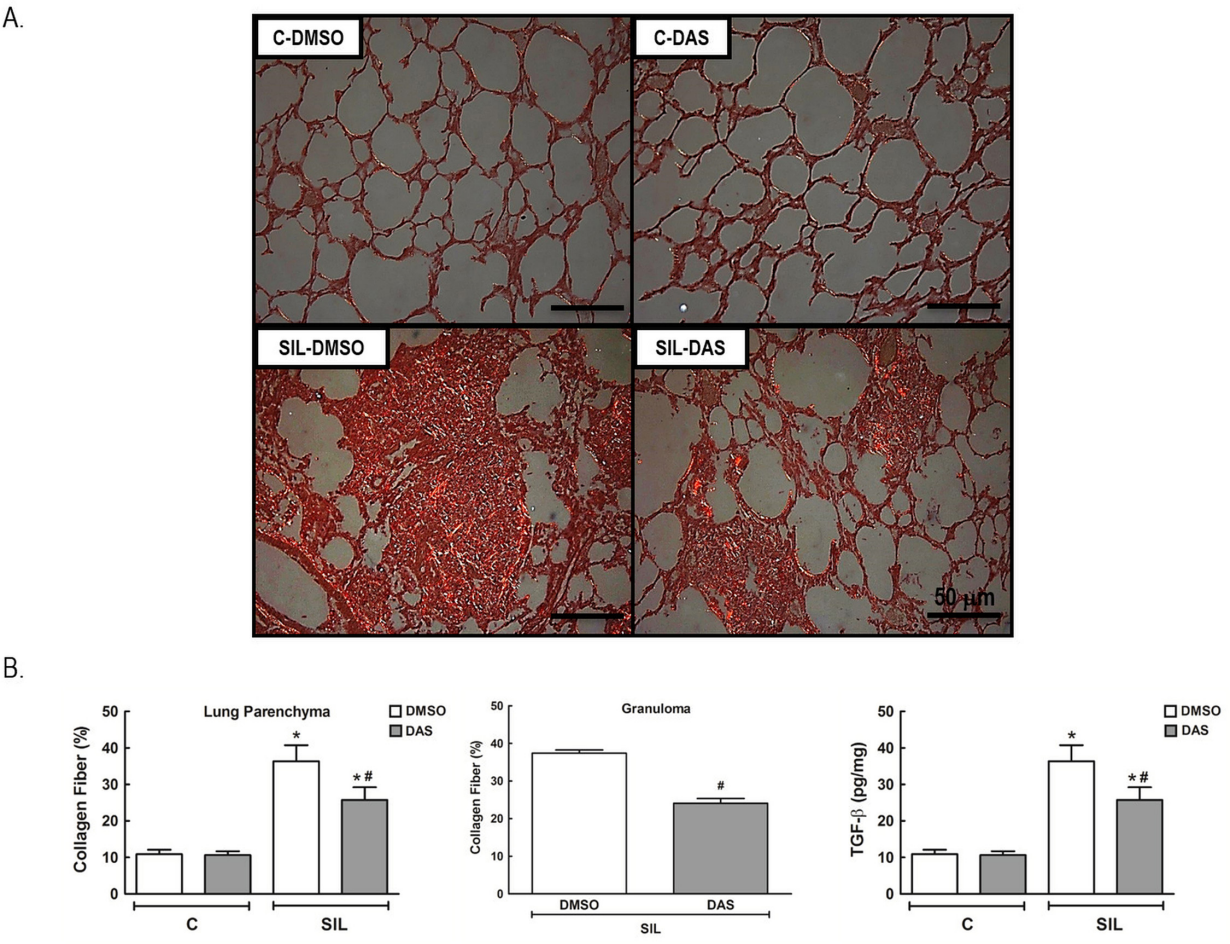
Fibrosis. (A) Representative photomicrographs (polarized light microscopy) of lung parenchyma, stained by Picrosirius red, of C-DMSO, C-DAS, SIL-DMSO, SIL-DAS. Collagen fibers are shown in orange. 100× magnification. Scale bars = 50 μm. (B) Collagen fiber content quantification in lung parenchyma and granuloma. Lung tissue protein levels of TGF-β quantified by ELISA. White bars: DMSO; gray bars: DAS. Values are means (± SD) of 8–9 animals per group. *Significantly different from C-DMSO group (*p* < 0.05). ^#^DMSO *vs*. DAS (*p <* 0.05).

The amount of collagen fibers and the level of TGF-β were increased in lung parenchyma and granuloma in SIL-DMSO compared to C-DMSO animals. Dasatinib led to reductions in collagen fiber deposition and TGF-β (Fig 7, Table G in S1 File).

### In Vitro Assays

**Fig 8:**
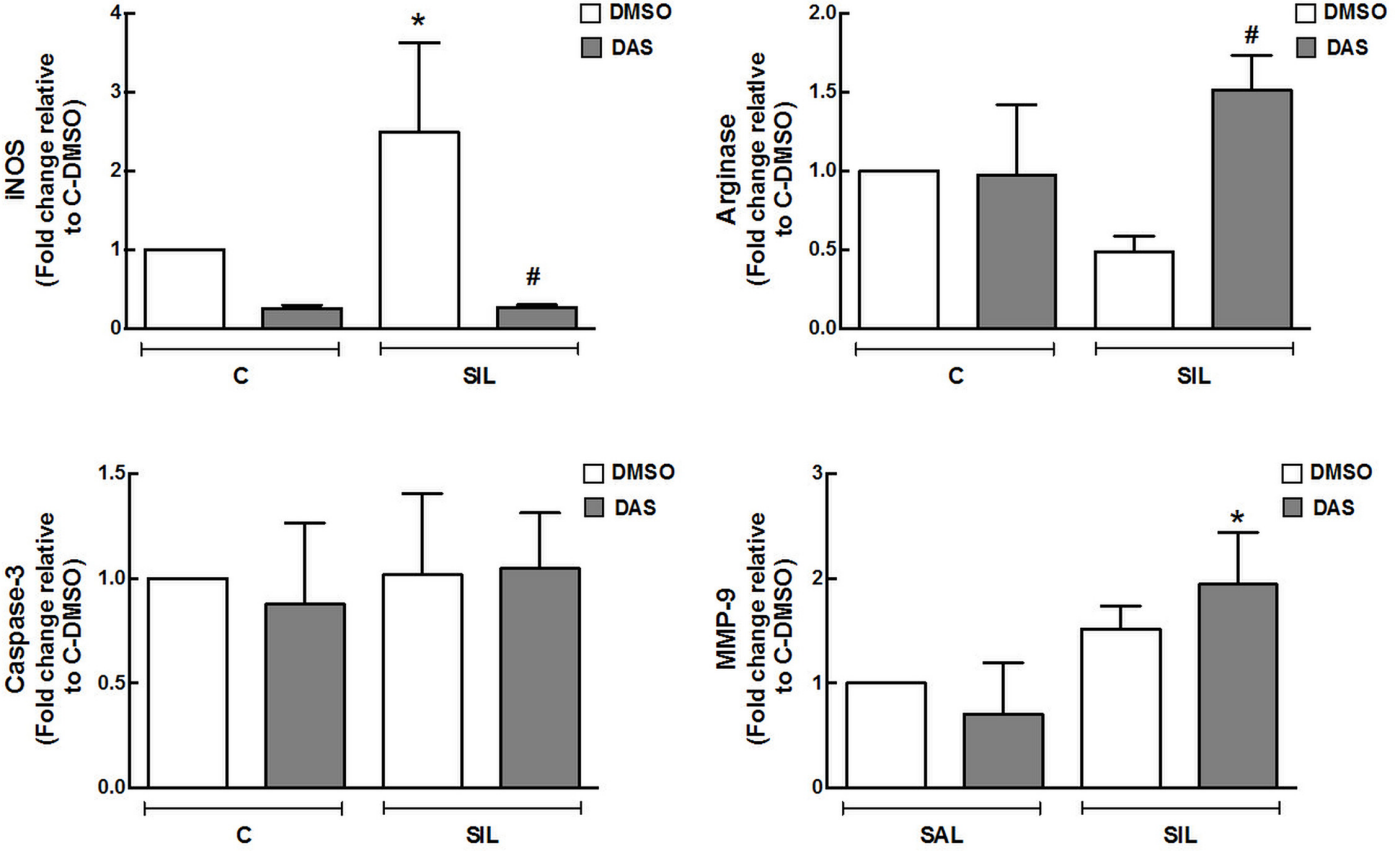
*In vitro* Assay. **RAW 264.7, a macrophage cell line, was incubated with orwithout silica oxide, and incubated for 24 hours with or without dasatinib.** mRNA levels of arginase, iNOS, MMP-9, caspase-3. White bars: DMSO; gray bars: DAS. Values are means (± SD), 3 wells per condition. *Significantly different from C-DMSO group (*p* < 0.05). ^#^DMSO *vs*. DAS (*p <* 0.05).

iNOS expression was increased in SIL-DMSO compared to C-DMSO animals. In SIL-DAS, arginase and MMP-9 expressions were increased compared to SIL-DMSO. No significant changes were observed in caspase-3 expression between groups (Fig 8, Table G in S1 File).

## Discussion

In the model of acute silicosis used herein, dasatinib improved lung mechanics and led to a reduction of fraction area of granuloma, neutrophils in lung tissue and granuloma, M1 macrophages in lung parenchyma and granuloma, fraction area of collapsed alveoli, collagen fiber content in lung parenchyma, protein levels of IL-1β, TNF-α, and TGF-β, and increased M2 macrophages in lung parenchyma and granuloma. *In vitro* studies showed that dasatinib led to reduced expression of iNOS and increased expression of arginase and MMP-9. To the best of our knowledge, this was the first study to evaluate the potential therapeutic effects of dasatinib on lung function, inflammation, and remodeling in experimental silicosis. The 1 mg/kg dose of dasatinib was chosen on the basis of pilot studies and studies in experimental endotoxin-induced acute lung injury conducted in our lab. The anti-fibrotic effects observed at this dose were similar to those observed at the higher dose (10 mg/kg). However, the higher dose led to further lung damage (data not shown).

The model of acute silicosis induced by a single exposure to crystalline silica used herein led to morphological and functional changes after 2 weeks which resembled human silicosis [2,19, 23]. Silicotic mice exhibited higher values of Est,L and ΔP2,L, which were associated with the presence of silicotic granulomas, alveolar collapse, thickening of alveolar septa, and inflammatory cell infiltration. The changes in ΔP1,L may correlate with an increased number of intrabronchial cells associated with lumen obstruction, in accordance with previous studies on silicotic mice [2,19, 23,24]. In the SIL-DMSO group, an increase in the number of macrophages and neutrophils, both in lung parenchyma and granuloma, was observed. Macrophages are the main cells involved in the pathophysiology of silicosis. Briefly, silica particles induce macrophage activation and lesion, triggering the release of metalloproteinases, free radicals, pro-inflammatory mediators such as IL-1β, TNF-α, and TGF-βthrough activation of nuclear factor (NF)-κB pathway [25,26], which is responsible for the recruitment of more inflammatory cells to the site of injury and are involved in lung fibrosis [2]. In order to evaluate whether DMSO might mitigate or induce further lung damage, another group of animals was treated with saline. No significant differences in lung mechanics and morphometry or collagen fiber content were observed between the DMSO and SAL groups (Tables A-C in S1 File); thus, we presented the data obtained from the DMSO group.

Protein tyrosine kinases have been demonstrated to play a crucial role in the inflammatory signaling pathways induced by silica. Evidence suggests that free radicals, generated by silica-mediated reactions or macrophage activation, trigger activation of several tyrosine kinases, which subsequently leads to dimerization and nuclear translocation of the pro-inflammatory transcription factor NF-κB [22]. Thus, dasatinib, an ATP-competitive protein tyrosine kinase inhibitor, when used at therapeutic concentrations, inhibits the activity of Abl, Bcr-Abl, Src-family kinases and several additional kinases, including Src and Btk family members, c-Kit, PDGFR, and Eph receptors [27]. Besides its effect on malignant cells, dasatinib also blocks certain functions of various hematopoietic cells by inhibiting T lymphocyte activation and proliferation [8], suppressing natural killer cell toxicity [9], blocking allergen-induced release of histamine in blood basophils [10], affecting platelet activation [11] and reducing neutrophil activation and chemotaxis [12,13]. In the present study, dasatinib reduced neutrophils in lung parenchyma and granuloma, which might be explained by the ability of the drug to block neutrophil adhesion and migration through the inhibition of Syk, ERK and the p38 MAPK [12,13], and reduced lung protein levels of the pro-inflammatory and chemoattractant mediators IL-1βand TNF-α. Moreover, these inflammatory changes may be related to the effect of dasatinib in reducing the number of M1 macrophages and increasing the number of M2 macrophages in lung parenchyma and granuloma of silicotic mice. It is well known that activation of NF-κB, which regulates genes controlling several physiological processes including the innate immune responses and inflammation [28], promotes polarization of macrophages toward the M1 phenotype [29]. Therefore, dasatinib might disrupt this pathway signaling, as NF-kB activation is Src tyrosine kinase-dependent in macrophages [22]. The M2 macrophage may help to resolve inflammation through high endocytic clearance abilities and production of trophic factors, as well as reduced pro-inflammatory cytokine secretion [30]. M2 macrophages also generate arginase-1, which suppresses inflammation by inhibiting production of pro-inflammatory nitric oxide [31]. Furthermore, M2 cells express the IL-1 receptor antagonist, which inhibits the effects of the pro-inflammatory cytokine IL-1, the mannose receptor, and chitinase–like 3 [32].

Regarding remodeling, there is great controversy on the role of M2 macrophages in fibrosis. M2 macrophages are increased in pulmonary fibrosis and are associated with fibrosis development [33, 34]. On the other hand, in the context of silicosis, Misson et al. addressed the question of whether lung fibrosis development is associated with M2 macrophages in a murine model of single intratracheal instillation of silica particles. By comparing the phenotype of pulmonary macrophages during the development of silica-induced lung fibrosis in C57BL/6 and BALB/c mice, the authors observed that the amplitude of Arg-1 mRNA up-regulation was not associated with the severity of lung fibrosis. Their data indicate that the establishment of a fibrotic process is not necessarily associated with M2 polarization in a murine silicosis model [35]. Furthermore, new evidence suggests that M2 macrophages may actually contribute to the resolution of fibrosis. Their presence during fibrosis, which has been observed in experiments, may be explained as a failing attempt to clear excess extracellular matrix. Mechanistic studies in models of liver fibrosis showed that M2 macrophages are not required for fibrosis development [36], and that M2 cells are important for fibrosis resolution in the liver [37,38]. Furthermore, it has been reported that a subset of adipose tissue macrophages exhibiting an M2 phenotype produce MMP-9, which in the kidney may contribute to attenuation of fibrotic lesions [39]. This correlates well with findings that uptake of extracellular matrix components appears to be mediated by M2 macrophages, as uptake of these components is mediated by different mannose receptors, which are known as M2 markers [40]. The conflicting roles described in the literature may be the result of difficulties in separating the effects of all existing subtypes of macrophages, since subsets are difficult to distinguish [33]. In our experiments, dasatinib increased the amount of M2 macrophages and reduced collagen deposition in lung parenchyma and granuloma. This phenomenon might be explained by the antifibrotic effects of M2 macrophages, but also by direct pharmacological inhibition of TK associated with pro-fibrotic receptors such as platelet-derived growth factor receptors (PDGFR) α and β, vascular endothelial growth factor receptors (VEGFR) 1, 2, and 3, fibroblast growth factor receptors (FGFR) 1, 2, and 3, and TGF- β.

Tyrosine kinase inhibitors are known to have antifibrotic effects. Nintedanib (formerly known as BIBF 1120) is an intracellular inhibitor that targets multiple tyrosine kinases. A phase 2 trial suggested that treatment with 150 mg of nintedanib twice-daily reduced lung-function decline and acute exacerbations in patients with idiopathic pulmonary fibrosis. However, several patients discontinued treatment because of adverse effects. Nintedanib was frequently associated with gastrointestinal disturbances such as diarrhea, elevated liver enzyme levels, and adverse events related to cardiac disorders, including ischemic heart disease [41]. Thus, despite some beneficial effects, nintedanib has a considerable adverse event profile. Dasatinib, a second-generation tyrosine kinase inhibitor, was chosen for our study because it is safe, presents potent antifibrotic effects, and has a lower cost [8]. Additionally, a potential therapeutic strategy for the treatment of silicosis would be to administer drugs that could induce the formation of ‘regulatory’-like macrophages at sites of inflammation. Dasatinib induces several hallmark features of ‘regulatory’-like macrophages. Treatment of macrophages with dasatinib increases production of IL-10 while suppressing production of TNF-α [42].

## Conclusions

Tissue fibrosis causes organ failure and death in patients with silicosis, and, to date, there are no clearly effective antifibrotic therapies available. As dasatinib has been demonstrated to reduce lung inflammation, minimize remodeling through inhibition of the pro-inflammatory cascade and stimulation of anti-inflammatory and antifibrotic cells, and improve lung mechanics, it may be an interesting option for the treatment of silicosis.

## Supporting Information

S1 File RAW DATA; Table A. Lung Mechanics (Raw Data). EST,L–Lung Static Elastance, ΔP1,L—Resistive Pressure, ΔP2,L -Viscoelastic Pressure. Control group (C) instilled with sterile saline and Silicosis group (SIL) instilled with silica particle. Fourteen days after disease induction, the animals were randomized to receive a solution of dimethyl sulfoxide (DMSO 1% in saline solution, 100 μL), dasatinib (DAS 1 mg/kg body weight in DMSO 1%, 100 μL) or saline (SAL, 100 μL). **Table B. Lung and granuloma morphometry and inflammation (Raw Data).** Granuloma Fraction (%),Granuloma Cellularity—Mononuclear Cells (%), Granuloma Cellularity—Neutrophils (%), Lung tissue protein levels of IL-1β Lung tissue protein levels of TNF-α Control group (C) instilled with sterile saline and Silicosis group (SIL) instilled with silica particle. Fourteen days after disease induction, the animals were randomized to receive a solution of dimethyl sulfoxide (DMSO 1% in saline solution, 100 μL), dasatinib (DAS 1 mg/kg body weight in DMSO 1%, 100 μL) or saline (SAL, 100 μL). **Table C. Lung Morphometry and Differential Cell Count (Raw Data).** Normal Alveoli (%), Collapsed Area (%), Hyperinflation (%), Neutrophils (%), Mononuclear Cells (%), Total Cells (%). Control Group (C) instilled with sterile saline and Silicosis group (SIL) instilled with silica particle. Fourteen days after disease induction, the animals were randomized to receive a solution of dimethylsulfoxide (DMSO 1% in saline solution, 100 μL), dasatinib (DAS 1mg/kg body weight in DMSO 1%, 100 μL) or saline (SAL, 100 μL). **Table D. Quantification of total macrophages (F4/80 positive cells—Raw Data).** F4/80 positive cells in lung parenchyma, F4/80 positive cells in silicotic granuloma. Control group (C) instilled with sterile saline and Silicosis group (SIL) instilled with silica particle. Fourteen days after disease induction, the animals were randomized to receive a solution of dimethyl sulfoxide (DMSO 1% in saline solution, 100 μL) or dasatinib (DAS 1 mg/kg body weight in DMSO 1%, 100 μL). **Table E. Quantification of subtype M1 macrophages (iNOS positive cells—Raw Data).** iNOS positive cells in lung parenchyma. iNOS positive cells in silicotic granuloma. Control group (C) instilled with sterile saline and Silicosis group (SIL) instilled with silica particle. Fourteen days after disease induction, the animals were randomized to receive a solution of dimethyl sulfoxide (DMSO 1% in saline solution, 100 μL) or dasatinib (DAS 1 mg/kg body weight in DMSO 1%, 100 μL). **Table F. Quantification of subtype M2 macrophages (Arginase positive cells—Raw Data).** Arginase positive cells in lung parenchyma, Arginase positive cells in silicotic granuloma. Control group (C) instilled with sterile saline and Silicosis group (SIL) instilled with silica particle. Fourteen days after disease induction, the animals were randomized to receive a solution of dimethyl sulfoxide (DMSO 1% in saline solution, 100 μL), dasatinib (DAS 1 mg/kg body weight in DMSO 1%, 100 μL). **Table G. Fibrosis. Collagen fiber content in lung parenchyma (Raw Data).** Collagen fiber content in silicotic granuloma. Control group (C) instilled with sterile saline and Silicosis group (SIL) instilled with silica particle. Fourteen days after disease induction, the animals were randomized to receive a solution of dimethyl sulfoxide (DMSO 1% in saline solution, 100 μL) or dasatinib (DAS 1 mg/kg body weight in DMSO 1%, 100 μL). **Table H. In vitro Assay (Raw Data).** mRNA levels of arginase, mRNA levels of iNOS, mRNA levels of MMP-9, mRNA levels of caspase-3. Control group (C) instilled with sterile saline and Silicosis group (SIL) instilled with silica particle. Fourteen days after disease induction, the animals were randomized to receive a solution of dimethyl sulfoxide (DMSO 1% in saline solution, 100 μL) or dasatinib (DAS 1 mg/kg body weight in DMSO 1%, 100 μL).
(DOCX)
