# Supplementary material for: Dasatinib Reduces Lung Inflammation and Fibrosis in Acute Experimental Silicosis
Source: PLoS One. 2016 Jan 20;11(1):e0147005. doi: 10.1371/journal.pone.0147005 (PMC4720427; doi:10.1371/journal.pone.0147005)
Supplement: S1 File — EST,L–Lung Static Elastance, ΔP1,L—Resistive Pressure, ΔP2,L -Viscoelastic Pressure. Control group (C) instilled with sterile saline and Silicosis group (SIL) instilled with silica particle. Fourteen days after disease induction, the animals were randomized to receive a solution of dimethyl sulfoxide (DMSO 1% in saline solution, 100 μL), dasatinib (DAS 1 mg/kg body weight in DMSO 1%, 100 μL) or saline (SAL, 100 μL). Table B. Lung and granuloma morphometry and inflammation (Raw Data). Granuloma Fraction (%),Granuloma Cellularity—Mononuclear Cells (%), Granuloma Cellularity—Neutrophils (%), Lung tissue protein levels of IL-1β Lung tissue protein levels of TNF-α Control group (C) instilled with sterile saline and Silicosis group (SIL) instilled with silica particle. Fourteen days after disease induction, the animals were randomized to receive a solution of dimethyl sulfoxide (DMSO 1% in saline solution, 100 μL), dasatinib (DAS 1 mg/kg body weight in DMSO 1%, 100 μL) or saline (SAL, 100 μL). Table C. Lung Morphometry and Differential Cell Count (Raw Data). Normal Alveoli (%), Collapsed Area (%), Hyperinflation (%), Neutrophils (%), Mononuclear Cells (%), Total Cells (%). Control Group (C) instilled with sterile saline and Silicosis group (SIL) instilled with silica particle. Fourteen days after disease induction, the animals were randomized to receive a solution of dimethylsulfoxide (DMSO 1% in saline solution, 100 μL), dasatinib (DAS 1mg/kg body weight in DMSO 1%, 100 μL) or saline (SAL, 100 μL). Table D. Quantification of total macrophages (F4/80 positive cells—Raw Data). F4/80 positive cells in lung parenchyma, F4/80 positive cells in silicotic granuloma. Control group (C) instilled with sterile saline and Silicosis group (SIL) instilled with silica particle. Fourteen days after disease induction, the animals were randomized to receive a solution of dimethyl sulfoxide (DMSO 1% in saline solution, 100 μL) or dasatinib (DAS 1 mg/kg body weight in DMSO 1%, 100 μL). Tabl [file pone.0147005.s001.docx]

| **Table A - Lung Mechanics** | |  |  |  |  |  |  |
| --- | --- | --- | --- | --- | --- | --- | --- |
|  |  |  |  |  |  |  |  |
| **EST,L – Lung Static Elastance** | | |  |  |  |  |  |
|  | **C-DMSO** | **C-DAS** | **SIL-DMSO** | **SIL-DAS** |  | **C-SAL** | **SIL-SAL** |
| **Animal 1** | 43,61 | 38,39 | 49,76 | 37,58 |  | 39,06 | 45,03 |
| **Animal 2** | 36,01 | 28,78 | 64,23 | 36,66 |  | 37,55 | 50,98 |
| **Animal 3** | 40,91 | 35,73 | 73,77 | 43,76 |  | 33,59 | 63,00 |
| **Animal 4** | 34,55 | 31,50 | 39,30 | 32,06 |  | 36,77 | 51,01 |
| **Animal 5** | 32,66 | 37,95 | 50,50 | 31,33 |  |  |  |
| **Animal 6** | 32,66 | 38,30 | 37,19 | 37,38 |  |  |  |
| **Animal 7** | 36,41 | 41,35 | 44,38 | 47,89 |  |  |  |
| **Animal 8** | 43,86 | 39,24 | 38,88 | 33,99 |  |  |  |
| **Animal 9** | 37,58 |  |  |  |  |  |  |
|  |  |  |  |  |  |  |  |
| **ΔP1,L - Resistive Pressure** | |  |  |  |  |  |  |
|  | **C-DMSO** | **C-DAS** | **SIL-DMSO** | **SIL-DAS** |  | **C-SAL** | **SIL-SAL** |
| **Animal 1** | 0,16901 | 0,19830 | 0,60564 | 0,18725 |  | 0,21957 | 1,14413 |
| **Animal 2** | 0,09184 | 0,11390 | 6,29571 | 0,44772 |  | 0,20347 | 1,01462 |
| **Animal 3** | 0,13630 | 0,12330 | 5,75096 | 0,70256 |  | 0,26223 | 1,18267 |
| **Animal 4** | 0,09103 | 0,05630 | 0,24662 | 0,77103 |  | 0,26564 | 1,09285 |
| **Animal 5** | 0,23751 | 0,27864 | 0,38296 | 0,56062 |  |  |  |
| **Animal 6** | 0,23751 | 0,26721 | 0,69120 | 1,23543 |  |  |  |
| **Animal 7** | 0,08755 | 0,18471 | 0,34074 | 0,45223 |  |  |  |
| **Animal 8** | 0,44688 | 0,21475 | 0,34238 | 0,47748 |  |  |  |
| **Animal 9** | 0,18720 |  |  |  |  |  |  |
|  |  |  |  |  |  |  |  |
| **ΔP2,L -Viscoelastic Pressure** | |  |  |  |  |  |  |
|  | **C-DMSO** | **C-DAS** | **SIL-DMSO** | **SIL-DAS** |  | **C-SAL** | **SIL-SAL** |
| **Animal 1** | 0,80916 | 0,75510 | 1,03865 | 0,76803 |  | 0,68661 | 1,11152 |
| **Animal 2** | 0,74395 | 0,66090 | 1,18535 | 0,79028 |  | 0,72554 | 1,10084 |
| **Animal 3** | 0,84860 | 0,80250 | 1,38487 | 0,81028 |  | 0,71523 | 0,84450 |
| **Animal 4** | 0,66941 | 0,73840 | 0,69631 | 0,89276 |  | 0,73000 | 0,92458 |
| **Animal 5** | 0,85238 | 0,91252 | 0,67990 | 0,81841 |  |  |  |
| **Animal 6** | 0,85238 | 0,84845 | 0,93935 | 0,77735 |  |  |  |
| **Animal 7** | 0,62136 | 0,83869 | 1,01878 | 0,83280 |  |  |  |
| **Animal 8** | 0,96825 | 0,76444 | 0,73481 | 0,89339 |  |  |  |
| **Animal 9** | 0,79570 |  |  |  |  |  |  |
|  |  |  |  |  |  |  |  |

| **Table B- Lung and granuloma morphometry and inflammation** | | | |  |  |  |  |
| --- | --- | --- | --- | --- | --- | --- | --- |
|  |  |  |  |  |  |  |  |
| **Granuloma Fraction (%)** | |  |  |  |  |  |  |
|  | **C-DMSO** | **C-DAS** | **SIL-DMSO** | **SIL-DAS** |  | **C-SAL** | **SIL-SAL** |
| **Animal 1** | - | - | 100,00 | 88,30 |  | - | 92,79 |
| **Animal 2** | - | - | 100,00 | 94,60 |  | - | 88,84 |
| **Animal 3** | - | - | 100,00 | 79,30 |  | - | 98,02 |
| **Animal 4** | - | - | 98,39 | 96,05 |  | - | 92,67 |
| **Animal 5** | - | - | 96,74 | 0,00 |  |  |  |
| **Animal 6** | - | - | 93,60 | 0,00 |  |  |  |
| **Animal 7** | - | - | 100,00 | 56,98 |  |  |  |
| **Animal 8** | - | - | 97,50 | 59,32 |  |  |  |
|  |  |  |  |  |  |  |  |
| **Granuloma Cellularity - Mononuclear Cells (%)** | | |  |  |  |  |  |
|  | **C-DMSO** | **C-DAS** | **SIL-DMSO** | **SIL-DAS** |  | **C-SAL** | **SIL-SAL** |
| **Animal 1** | - | - | 35,12 | 31,31 |  | - | 27,63 |
| **Animal 2** | - | - | 29,79 | 22,05 |  | - | 33,95 |
| **Animal 3** | - | - | 36,40 | 24,50 |  | - | 28,33 |
| **Animal 4** | - | - | 27,30 | 35,90 |  | - | 37,08 |
| **Animal 5** | - | - | 29,79 | 30,40 |  |  |  |
| **Animal 6** | - | - | 24,26 | 27,90 |  |  |  |
| **Animal 7** | - | - | 30,27 | 35,20 |  |  |  |
| **Animal 8** | - | - | 29,20 | 37,80 |  |  |  |
|  |  |  |  |  |  |  |  |
|  |  |  |  |  |  |  |  |
|  |  |  |  |  |  |  |  |
|  |  |  |  |  |  |  |  |
| **Granuloma Cellularity - Neutrophils (%)** | | |  |  |  |  |  |
|  | **C-DMSO** | **C-DAS** | **SIL-DMSO** | **SIL-DAS** |  | **C-SAL** | **SIL-SAL** |
| **Animal 1** | - | - | 2,68 | 3,65 |  | - | 6,90 |
| **Animal 2** | - | - | 6,08 | 5,25 |  | - | 4,63 |
| **Animal 3** | - | - | 6,10 | 5,20 |  | - | 7,44 |
| **Animal 4** | - | - | 3,90 | 2,60 |  | - | 7,18 |
| **Animal 5** | - | - | 6,08 | 2,80 |  |  |  |
| **Animal 6** | - | - | 6,42 | 3,20 |  |  |  |
| **Animal 7** | - | - | 6,00 | 1,70 |  |  |  |
| **Animal 8** | - | - | 5,32 | 3,29 |  |  |  |
|  |  |  |  |  |  |  |  |
| **Lung tissue protein levels of IL-1β** | | |  |  |  |  |  |
|  | **C-DMSO** | **C-DAS** | **SIL-DMSO** | **SIL-DAS** |  |  |  |
| **Animal 1** | 87,65 | 24,29 | 671,07 | 242,36 |  |  |  |
| **Animal 2** | 110,94 | 13,41 | 543,05 | 250,37 |  |  |  |
| **Animal 3** | 51,44 | 25,95 | 526,07 | 145,29 |  |  |  |
| **Animal 4** | 96,64 | 19,39 | 850,58 | 390,04 |  |  |  |
| **Animal 5** | 40,96 | 21,83 | 79,61 | 399,27 |  |  |  |
| **Animal 6** | 81,44 | 18,76 | 596,27 | 296,51 |  |  |  |
| **Animal 7** | 89,98 | 34,75 | 623,94 | 305,48 |  |  |  |
| **Animal 8** | 63,94 | 22,62 | 484,82 | 392,73 |  |  |  |
| **Animal 9** | 78,28 |  |  | 234,46 |  |  |  |
|  |  |  |  |  |  |  |  |
|  |  |  |  |  |  |  |  |
|  |  |  |  |  |  |  |  |
|  |  |  |  |  |  |  |  |
|  |  |  |  |  |  |  |  |
| **Lung tissue protein levels of TNF-α** | | |  |  |  |  |  |
|  | **C-DMSO** | **C-DAS** | **SIL-DMSO** | **SIL-DAS** |  |  |  |
| **Animal 1** | 30,22 | 15,19 | 54,25 | 23,23 |  |  |  |
| **Animal 2** | 27,55 | 7,49 | 56,27 | 21,26 |  |  |  |
| **Animal 3** | 23,14 | 14,33 | 41,60 | 13,70 |  |  |  |
| **Animal 4** | 26,49 | 11,66 | 12,55 | 22,84 |  |  |  |
| **Animal 5** | 18,09 | 18,52 | 49,32 | 30,00 |  |  |  |
| **Animal 6** | 24,69 | 12,62 | 18,66 | 19,08 |  |  |  |
| **Animal 7** | 30,26 | 13,03 | 43,54 | 17,51 |  |  |  |
| **Animal 8** | 26,89 | 13,26 | 39,50 | 20,47 |  |  |  |
| **Animal 9** | 24,32 |  |  | 16,52 |  |  |  |
|  |  |  |  |  |  |  |  |

| **Table C- Lung Morphometry and Differential Cell Count** | | | |  |  |  |  |
| --- | --- | --- | --- | --- | --- | --- | --- |
|  |  |  |  |  |  |  |  |
| **Nomal Alveoli(%)** | |  |  |  |  |  |  |
|  | **C-DMSO** | **C-DAS** | **SIL-DMSO** | **SIL-DAS** |  | **C-SAL** | **SIL-SAL** |
| **Animal 1** | 96,60 | 98,56 | 68,26 | 84,06 |  | 96,99 | 74,32 |
| **Animal 2** | 84,82 | 97,50 | 81,21 | 84,30 |  | 96,93 | 78,16 |
| **Animal 3** | 95,66 | 98,46 | 71,93 | 90,78 |  | 99,68 | 73,24 |
| **Animal 4** | 88,84 | 87,49 | 71,52 | 85,93 |  | 96,84 | 72,13 |
| **Animal 5** | 96,40 | 98,83 | 76,24 | 92,17 |  |  |  |
| **Animal 6** | 87,01 | 88,40 | 71,24 | 90,66 |  |  |  |
| **Animal 7** | 97,08 | 91,06 | 75,10 | 84,67 |  |  |  |
| **Animal 8** | 87,33 | 84,69 | 66,58 | 93,93 |  |  |  |
| **Animal 9** |  |  |  |  |  |  |  |
|  |  |  |  |  |  |  |  |
| **Collapsed Area (%)** | |  |  |  |  |  |  |
|  | **C-DMSO** | **C-DAS** | **SIL-DMSO** | **SIL-DAS** |  | **C-SAL** | **SIL-SAL** |
| **Animal 1** | 3,40 | 1,44 | 28,16 | 4,81 |  | 3,01 | 25,68 |
| **Animal 2** | 15,18 | 2,50 | 18,79 | 0,00 |  | 3,07 | 21,84 |
| **Animal 3** | 4,34 | 1,54 | 22,95 | 0,00 |  | 0,32 | 26,76 |
| **Animal 4** | 11,16 | 12,51 | 28,48 | 0,00 |  | 3,16 | 27,87 |
| **Animal 5** | 3,60 | 1,17 | 23,76 | 0,00 |  |  |  |
| **Animal 6** | 12,99 | 11,60 | 28,76 | 0,00 |  |  |  |
| **Animal 7** | 2,92 | 8,94 | 24,18 | 0,73 |  |  |  |
| **Animal 8** | 12,67 | 15,31 | 33,42 | 0,00 |  |  |  |
| **Animal 9** |  |  |  |  |  |  |  |
|  |  |  |  |  |  |  |  |
|  |  |  |  |  |  |  |  |
| **Hyperinflation (%)** | |  |  |  |  |  |  |
|  | **C-DMSO** | **C-DAS** | **SIL-DMSO** | **SIL-DAS** |  | **C-SAL** | **SIL-SAL** |
| **Animal 1** | 0,00 | 0,00 | 3,59 | 11,13 |  | 0,00 | 0,00 |
| **Animal 2** | 0,00 | 0,00 | 0,00 | 15,70 |  | 0,00 | 0,00 |
| **Animal 3** | 0,00 | 0,00 | 5,12 | 9,22 |  | 0,00 | 0,00 |
| **Animal 4** | 0,00 | 0,00 | 0,00 | 14,07 |  | 0,00 | 0,00 |
| **Animal 5** | 0,00 | 0,00 | 0,00 | 7,83 |  |  |  |
| **Animal 6** | 0,00 | 0,00 | 0,00 | 9,34 |  |  |  |
| **Animal 7** | 0,00 | 0,00 | 0,72 | 14,60 |  |  |  |
| **Animal 8** | 0,00 | 0,00 | 0,00 | 6,07 |  |  |  |
| **Animal 9** |  |  |  |  |  |  |  |
|  |  |  |  |  |  |  |  |
| **Neutrophils (%)** | |  |  |  |  |  |  |
|  | **C-DMSO** | **C-DAS** | **SIL-DMSO** | **SIL-DAS** |  | **C-SAL** | **SIL-SAL** |
| **Animal 1** | 3,04 | 6,71 | 4,43 | 2,00 |  | 1,98 | 8,42 |
| **Animal 2** | 3,43 | 3,71 | 4,80 | 4,01 |  | 0,48 | 5,41 |
| **Animal 3** | 1,80 | 3,61 | 3,86 | 5,16 |  | 5,31 | 3,30 |
| **Animal 4** | 2,33 | 3,00 | 2,36 | 2,86 |  | 2,37 | 12,92 |
| **Animal 5** | 3,50 | 3,58 | 8,16 | 5,36 |  |  |  |
| **Animal 6** | 3,04 | 4,91 | 2,43 | 3,25 |  |  |  |
| **Animal 7** | 3,12 | 4,52 | 11,88 | 3,87 |  |  |  |
| **Animal 8** |  | 5,09 |  | 4,64 |  |  |  |
|  |  |  |  |  |  |  |  |
|  |  |  |  |  |  |  |  |
|  |  |  |  |  |  |  |  |
|  |  |  |  |  |  |  |  |
|  |  |  |  |  |  |  |  |
|  |  |  |  |  |  |  |  |
| **Mononuclear Cells (%)** | |  |  |  |  |  |  |
|  | **C-DMSO** | **C-DAS** | **SIL-DMSO** | **SIL-DAS** |  | **C-SAL** | **SIL-SAL** |
| **Animal 1** | 17,62 | 22,98 | 29,45 | 26,29 |  | 17,14 | 37,60 |
| **Animal 2** | 16,41 | 27,15 | 21,32 | 18,00 |  | 18,20 | 28,80 |
| **Animal 3** | 12,31 | 17,21 | 42,89 | 25,80 |  | 17,29 | 36,50 |
| **Animal 4** | 20,41 | 15,30 | 37,19 | 32,46 |  | 18,37 | 28,80 |
| **Animal 5** | 22,29 | 16,05 | 36,72 | 27,63 |  |  |  |
| **Animal 6** | 17,62 | 22,78 | 29,26 | 29,19 |  |  |  |
| **Animal 7** | 27,34 | 24,51 | 25,03 | 28,20 |  |  |  |
| **Animal 8** |  | 22,10 |  | 28,21 |  |  |  |
|  |  |  |  |  |  |  |  |
| **Total Cells (%)** | |  |  |  |  |  |  |
|  | **C-DMSO** | **C-DAS** | **SIL-DMSO** | **SIL-DAS** |  | **C-SAL** | **SIL-SAL** |
| **Animal 1** | 20,66 | 29,69 | 33,87 | 28,30 |  | 19,12 | 46,02 |
| **Animal 2** | 19,84 | 30,86 | 26,11 | 22,01 |  | 18,68 | 34,21 |
| **Animal 3** | 14,11 | 20,82 | 46,75 | 30,95 |  | 22,60 | 39,80 |
| **Animal 4** | 22,74 | 18,29 | 39,55 | 35,32 |  | 20,74 | 41,72 |
| **Animal 5** | 25,80 | 19,63 | 44,88 | 32,99 |  |  |  |
| **Animal 6** | 20,66 | 27,69 | 31,69 | 32,44 |  |  |  |
| **Animal 7** | 30,46 | 29,02 | 36,92 | 32,08 |  |  |  |
| **Animal 8** |  | 27,19 |  | 32,86 |  |  |  |

| **Table D - Quantification of total macrophages (F4/80 positive cells).** | | | |  |  |  |  |
| --- | --- | --- | --- | --- | --- | --- | --- |
|  |  |  |  |  |  |  |  |
| **F4/80 positive cells in lung parenchyma** | | |  |  |  |  |  |
|  | **C-DMSO** | **C-DAS** | **SIL-DMSO** | **SIL-DAS** |  |  |  |
| **Animal 1** | 2,4 | 5,7 | 21,1 | 12,4 |  |  |  |
| **Animal 2** | 1,9 | 4,8 | 45,1 | 6,3 |  |  |  |
| **Animal 3** | 1,6 | 0,8 | 9,5 | 11,4 |  |  |  |
| **Animal 4** | 3,1 | 3,6 | 35,7 | 24,2 |  |  |  |
| **Animal 5** | 2,2 | 2,5 | 15,0 | 13,0 |  |  |  |
|  |  |  |  |  |  |  |  |
| **F4/80 positive cells in silicotic granuloma** | | |  |  |  |  |  |
|  | **C-DMSO** | **C-DAS** | **SIL-DMSO** | **SIL-DAS** |  |  |  |
| **Animal 1** | - | - | 22,2 | 18,3 |  |  |  |
| **Animal 2** | - | - | 24,6 | 23,5 |  |  |  |
| **Animal 3** | - | - | 19,4 | 17,3 |  |  |  |
| **Animal 4** | - | - | 28,3 | 21,4 |  |  |  |
| **Animal 5** | - | - | 23,6 | 19,9 |  |  |  |
|  |  |  |  |  |  |  |  |
|  |  |  |  |  |  |  |  |

| **Table E - Quantification of subtype M1 macrophages (iNOS positive cells).** | | | | |  |  |  |
| --- | --- | --- | --- | --- | --- | --- | --- |
|  |  |  |  |  |  |  |  |
| **iNOS positive cells in lung parenchyma** | | |  |  |  |  |  |
|  | **C-DMSO** | **C-DAS** | **SIL-DMSO** | **SIL-DAS** |  |  |  |
| **Animal 1** | 0,0 | 1,0 | 10,0 | 1,0 |  |  |  |
| **Animal 2** | 0,0 | 0,0 | 11,0 | 0,5 |  |  |  |
| **Animal 3** | 0,3 | 0,0 | 12,0 | 1,2 |  |  |  |
| **Animal 4** | 0,4 | 0,0 | 13,0 | 0,4 |  |  |  |
| **Animal 5** | 0,5 | 0,3 | 11,0 | 0,7 |  |  |  |
| **Animal 6** | 0,7 | 0,2 | 10,0 | 1,2 |  |  |  |
| **Animal 7** | 0,8 |  | 9,0 |  |  |  |  |
| **Animal 8** |  |  | 11,0 |  |  |  |  |
|  |  |  |  |  |  |  |  |
| **iNOS positive cells in silicotic granuloma** | | |  |  |  |  |  |
|  | **C-DMSO** | **C-DAS** | **SIL-DMSO** | **SIL-DAS** |  |  |  |
| **Animal 1** | - | - | 30,0 | 0,4 |  |  |  |
| **Animal 2** | - | - | 32,0 | 1,0 |  |  |  |
| **Animal 3** | - | - | 33,0 | 1,2 |  |  |  |
| **Animal 4** | - | - | 35,0 | 2,2 |  |  |  |
| **Animal 5** | - | - | 34,0 | 1,5 |  |  |  |
| **Animal 6** | - | - | 30,0 | 1,0 |  |  |  |
| **Animal 7** | - | - | 25,0 |  |  |  |  |
| **Animal 8** | - | - | 31,0 |  |  |  |  |
|  |  |  |  |  |  |  |  |
|  |  |  |  |  |  |  |  |

| **Table F - Quantification of subtype M2 macrophages (Arginase positive cells).** | | | | |  |  |  |
| --- | --- | --- | --- | --- | --- | --- | --- |
|  |  |  |  |  |  |  |  |
| **Arginase positive cells in lung parenchyma** | | |  |  |  |  |  |
|  | **C-DMSO** | **C-DAS** | **SIL-DMSO** | **SIL-DAS** |  |  |  |
| **Animal 1** | 1,2 | 1,0 | 2,5 | 3,6 |  |  |  |
| **Animal 2** | 0,0 | 1,6 | 1,9 | 4,5 |  |  |  |
| **Animal 3** | 1,3 | 0,8 | 1,7 | 5,9 |  |  |  |
| **Animal 4** | 0,9 | 0,7 | 1,4 | 1,1 |  |  |  |
| **Animal 5** | 1,3 | 1,3 | 1,1 | 5,8 |  |  |  |
| **Animal 6** | 1,5 | 1,1 | 2,9 | 4,2 |  |  |  |
| **Animal 7** | 1,2 |  | 3,3 |  |  |  |  |
| **Animal 8** |  |  | 2,7 |  |  |  |  |
|  |  |  |  |  |  |  |  |
| **Arginase positive cells in silicotic granuloma** | | |  |  |  |  |  |
|  | **C-DMSO** | **C-DAS** | **SIL-DMSO** | **SIL-DAS** |  |  |  |
| **Animal 1** | - | - | 2,0 | 8,0 |  |  |  |
| **Animal 2** | - | - | 3,0 | 9,0 |  |  |  |
| **Animal 3** | - | - | 4,0 | 6,9 |  |  |  |
| **Animal 4** | - | - | 2,0 | 8,2 |  |  |  |
| **Animal 5** | - | - | 0,9 | 9,1 |  |  |  |
| **Animal 6** | - | - | 2,0 | 9,8 |  |  |  |
| **Animal 7** | - | - | 2,9 |  |  |  |  |
| **Animal 8** | - | - | 2,1 |  |  |  |  |
|  |  |  |  |  |  |  |  |

|  |  |  |  |  |  |  |  |
| --- | --- | --- | --- | --- | --- | --- | --- |
| **Table G - Fibrosis** | |  |  |  |  |  |  |
|  |  |  |  |  |  |  |  |
| **Collagen fiber content in lung parenchyma** | | |  |  |  |  |  |
|  | **C-DMSO** | **C-DAS** | **SIL-DMSO** | **SIL-DAS** |  |  |  |
| **Animal 1** | 10,00 | 11,00 | 32,00 | 24,00 |  |  |  |
| **Animal 2** | 11,00 | 12,00 | 32,00 | 28,00 |  |  |  |
| **Animal 3** | 12,00 | 10,00 | 36,00 | 24,00 |  |  |  |
| **Animal 4** | 13,00 | 9,00 | 34,00 | 31,33 |  |  |  |
| **Animal 5** | 11,00 | 10,00 | 37,19 | 21,00 |  |  |  |
| **Animal 6** | 10,00 | 10,00 | 44,38 | 27,89 |  |  |  |
| **Animal 7** | 9,00 | 11,00 | 38,88 | 23,99 |  |  |  |
| **Animal 8** | 11,00 | 12,00 |  |  |  |  |  |
|  |  |  |  |  |  |  |  |
| **Collagen fiber content in silicotic granuloma** | | |  |  |  |  |  |
|  | **C-DMSO** | **C-DAS** | **SIL-DMSO** | **SIL-DAS** |  |  |  |
| **Animal 1** | - | - | 35,12 | 21,31 |  |  |  |
| **Animal 2** | - | - | 39,79 | 22,05 |  |  |  |
| **Animal 3** | - | - | 36,40 | 24,50 |  |  |  |
| **Animal 4** | - | - | 37,30 | 25,90 |  |  |  |
| **Animal 5** | - | - | 39,79 | 30,40 |  |  |  |
| **Animal 6** | - | - | 34,26 | 27,90 |  |  |  |
| **Animal 7** | - | - | 39,20 | 25,20 |  |  |  |
| **Animal 8** | - | - |  | 17,80 |  |  |  |
| **Animal 9** |  |  |  | 21,53 |  |  |  |
|  |  |  |  |  |  |  |  |
| **Table H- In vitro Assay** | |  |  |  |  |  |  |
|  |  |  |  |  |  |  |  |
| **mRNA levels of arginase** | |  |  |  |  |  |  |
|  | **C-DMSO** | **C-DAS** | **SIL-DMSO** | **SIL-DAS** |  |  |  |
| **Sample1** | 1,0000 | 1,6029 | 0,5783 | 1,7777 |  |  |  |
| **Sample2** | 1,0000 | 0,5783 | 0,5548 | 1,4142 |  |  |  |
| **Sample3** | 1,0000 | 0,7423 | 0,3635 | 1,6021 |  |  |  |
| **Sample4** | 1,0000 | 0,9624 | 0,4506 | 1,2746 |  |  |  |
|  |  |  |  |  |  |  |  |
| **mRNA levels of iNOS** | |  |  |  |  |  |  |
|  | **C-DMSO** | **C-DAS** | **SIL-DMSO** | **SIL-DAS** |  |  |  |
| **Sample1** | 1,0000 | 0,2197 | 1,1837 | 0,2558 |  |  |  |
| **Sample2** | 1,0000 | 0,2420 | 2,0668 | 0,2919 |  |  |  |
| **Sample3** | 1,0000 | 0,3128 | 3,8606 | 0,2878 |  |  |  |
| **Sample4** | 1,0000 | 0,2371 | 2,8546 | 0,2137 |  |  |  |
|  |  |  |  |  |  |  |  |
| **mRNA levels of MMP-9** | |  |  |  |  |  |  |
|  | **C-DMSO** | **C-DAS** | **SIL-DMSO** | **SIL-DAS** |  |  |  |
| **Sample1** | 1,0000 | 0,3128 | 1,7777 | 1,7777 |  |  |  |
| **Sample2** | 1,0000 | 0,2371 | 1,4142 | 1,4142 |  |  |  |
| **Sample3** | 1,0000 | 1,1837 | 1,6021 | 2,6021 |  |  |  |
| **Sample4** | 1,0000 | 1,0668 | 1,2746 | 1,9746 |  |  |  |
|  |  |  |  |  |  |  |  |
|  |  |  |  |  |  |  |  |
|  |  |  |  |  |  |  |  |
|  |  |  |  |  |  |  |  |
|  |  |  |  |  |  |  |  |
|  |  |  |  |  |  |  |  |
| **mRNA levels of caspase-3** | |  |  |  |  |  |  |
|  | **C-DMSO** | **C-DAS** | **SIL-DMSO** | **SIL-DAS** |  |  |  |
| **Sample1** | 1,0000 | 0,3128 | 0,7777 | 0,7777 |  |  |  |
| **Sample2** | 1,0000 | 0,9371 | 1,4142 | 1,4142 |  |  |  |
| **Sample3** | 1,0000 | 1,1837 | 0,6021 | 1,0214 |  |  |  |
| **Sample4** | 1,0000 | 1,0668 | 1,2746 | 0,9746 |  |  |  |
|  |  |  |  |  |  |  |  |
|  |  |  |  |  |  |  |  |
|  |  |  |  |  |  |  |  |
|  |  |  |  |  |  |  |  |
|  |  |  |  |  |  |  |  |
|  |  |  |  |  |  |  |  |
|  |  |  |  |  |  |  |  |
|  |  |  |  |  |  |  |  |
|  |  |  |  |  |  |  |  |
